# Cases in Practice

**Published:** 1855-01

**Authors:** D. L. McGugin

**Affiliations:** Prof. Physiology and Pathology in Iowa Medical Department


### CASES IN PRACTICE.

BY D. L. MCGUGIN, M. D.,

Prof. Physiology and Pathology in Iowa Medical Department.

CASE I.—Mrs. P——, pregnant with her eighth child, had been a sufferer from dysentery for the period of sixteen days, at the end of which time I was consulted. I found her in such extremity of suffering, that she could not give me a satisfactory history of her case. She had suffered much from dysenteric discharges, and the attendants stated that during the above period, a number of times every 24 hours, the paroxysms of suffering were as great as the aggravated character which they then presented. I found her tossing, and rolling over the bed, in the most indescribable torture, evidently paroxysmal, but the intermissions short and incomplete. Although a woman of full habit, her pulse was frequent and feeble, numbering 140 to the minute. The tongue was slightly furred, and the expression of her countenance was one of great anxiety. She had slept but little during the above period; there had been distressing thirst for the past few days, and all her symptoms indicated great exhaustion. The dysenteric discharges were not remarkably frequent, nor were they of a character indicating much lesion of the lower bowels. The character of the suffering indicated to me that labor was then in progress, and the history of her case led to the belief that this had been the cause of her suffering for many days, and perhaps from the first, as she was strongly impressed with the opinion, that her term closed with the advent of her suffering. The "touch" discovered the os uteri dilated and dilatable, but looking backward toward the sacrum, and laterally to the right iliac fossa. I concluded at once that here was an anterior and lateral obliquity. An examination into the form and situation of the abdominal tumor, showed that the position of the uterus was to the left, and anteriorly, and also that there was very great laxity of the abdominal walls. The tumor was unusually large, nor did the examination reveal that the usual distension was owing in any great degree to over accumulation of liquor amnii. The fibres were contracting somewhat firmly, and revealed to a certain extent the form and position of the fœtus. This would not have been obtained, had there been a superabundance of the waters. The fœtus was living, as the pulsation of the fœtal heart was plainly perceptible.

The indication was now plain enough, and a proceeding was adopted in consonance. The change of position of the patient upon her back, and the adjustment of a broad roller over the abdomen with some firmness, were but the work of a moment. The bandage over the abdomen was to supply the requisite support which the abdominal parietes, by their extreme laxity, refused to afford. In a short time the pains increased in force, the propulsive effect was made evident, and in half an hour a healthy child of twelve pounds was born. In a few days the dysentery ceased under remedies, and she continued to improve.

This woman was doubtless in labor for some time, but the unavailing propulsive efforts were followed by a surrender of the uterine fibres after each, to be renewed after a regular period to another fruitless trial. Thus it continued, and thus it would have continued, unless accident had placed the uterus in a situation and relation to the axis of the pelvic cavity, or until her strength would have been exhausted, and the fœtus not yet delivered. This case is suggestive of a wise caution. In protracted labors we should enquire into all the particulars, with the view to the discovery of "all and singular" the causes arresting the labor in any of its stages. As already intimated, there was not half the usual quantity of the liquor amnii, the placenta followed promptly, and the uterus contracted readily and fully.

There is another fact or two worthy of notice. The dysentery existed during the sixteen days above mentioned, but the paroxysms of suffering which appeared from the first, were not always an attendant circumstance upon the fruitless and unavailing attempts to evacuate the bowels. Below the situation of the os, the rectum appeared congested, thickened, and highly sensitive, when even gently pressed upon. Now what agency had the local pressure upon this portion of the rectum in the production of these repeated and painful peristaltic movements? My opinion is that this unusual, special, and severe compression was the entire cause, for following the delivery, in a few hours, there were several large scybalic discharges, and these continued for some hours at intervals, after which the dysenteric symptoms ceased. It were useless here to refer to the fact of the retention as one cause of irritation, the special compression amounting probably to almost an obliteration of the intestinal tube at that spot in its length as the chief, and how much of irritation was produced by the highly vascular condition of the rectum below the pressure. In this case one difficulty was produced by and followed another, each of which was in itself sufficiently serious, but both subsiding upon the removal of the first.

Case ii.—Mrs. S——, nursing a healthy vigorous child, who had recently been gifted with a pair of fine incisors, and who after repeated trials of their peculiar qualities, had succeeded in producing an abrasion near the nipple, and in a spirit of *mal prepense*, had continued to keep up the abrasion, and was attacked with a severe chill, followed by a most extravagant paroxysm of fever, attended with great heat of skin, thirst inappeasable, and wild delirium.—The breasts were examined, when this abrasion was detected, beyond which and extending to the axilla, a red blush was seen, an evident tumefaction and excessive tenderness; a manifest case of milk fever, as the reader will readily perceive. A brisk cathartic was given, the breast was elevated and secured so as to relieve the milk tubes of the mammæ in the part above indicated, the affected part painted freely with the alcoholic tinc. iodine, an emollient poultice applied over all, directed liquor morph. comp. gutta xx, after the action of the cathartic and the subsidence of the fever.

Next morning found her without fever, mammæ less painful, cathartic had acted freely, the anodyne had its desired effect, and she continued to improve without any further difficulty with the breast; since then has had several paroxysms of the same kind and character, and relieved by the same means. The abrasion was the cause each time of the paroxysm, by the irritation extending along the milk tubes and arresting the flow of the milk; but care was exercised to encourage the discharge by frequent exhaustion by nursing. A reluctance to the use of any application to the abraded surface, permitted it to continue a source of irritation—the collodion having once produced some painful sensations.

CASE III.—Miss J——, had during the summer suffered from a severe attack of cholera, which was followed by fever of a low type, attended with gastric symptoms. The fever having subsided, the gastric symptoms remained, a serious impediment to her recovery. The appetite was not only impaired, but entirely lost, and the stomach so irritable that the bare mention of food awakened associations which disturbed the repose of the stomach, and eructations would at once occur. These occurred when the blandest food was taken. The tongue was red and contracted at tip, the face and lips pale, pulse 120 to 140—great emaciation and prostration, extremities often cold, bowels constipated, great thirst, restlessness, great irritability of mind and despondency. Every means was used internally and externally to relieve the irritability aud restore the tone of the stomach, an enumeration of which would extend this report to a needless length. Suffice it now for our purpose to say, that it was apparent that unless something was devised to meet and control this local difficulty, inanition and death must soon follow.

In this dilemma the Trisnitrate of Bismuth, with the compound aromatic powder, was prescribed with the happiest results, as she began to convalesce after the first administration. The following was the formula:

℞ Trisnitrate Bismuth, grs. iij.,
Pulv. Arom., Com. grs. ij.
M

To be given three times a day. The eructations ceased, the appetite returned, and with the aid of gentle tonics and stimulants, she began rapidly to improve, and continues to convalesce. This is not the first or only case in which we have found this drug of value in the treatment of diseases of the stomach, attended with a loss of tone.

CASE IV.—Mrs. B——, a young lady married for some years, but had never conceived, labored under dysmenorrhœa, and afterward severe leucorrhœa, both of which increased in time. The speculum revealed a deep fissure in the posterior lip of the os uteri, with an hypertrophied condition of the neck of the uterus and of the lips of the os. The fissure was twice touched with a solution of the iodide zinc, 1 scrup. to the ounce of distilled water, and then after adding double the quantity of water, it was afterwards used as an injection once a day.

There was an anœmic condition attending, for which the syrup of ferri iodide was used three times each day persistently. The last monthly catamenia was without pain, the first for several years—the leucorrhœa almost entirely ceased, the lips showed a better supply of red corpuscles in the blood, and the spirits a great improvement. A tactile enquiry shows a decided amendment in the condition of the neck and structure at the os. The iodine zinc has succeeded well in our observation in a cure of purely vaginal leucorrhœa ; we think it has strong claims to favor in these troublesome but frequent afflictions.

CASE V.—Mr. E——, of this city, complaining of tooth-ache, concluded upon its extraction, and applied for that purpose to a surgeon dentist, who, before he had commenced an examination of the condition of the tooth, noticed that the patient exhibited some irregular motions of the limbs and in the muscles of the face and neck. The dental surgeon regarded the phenomena as those of chorea, as the feet moved in various directions, and the hands and arms as if turning a crank. The jaws were however soon closed, so much so as to make it difficult to indicate that he desired medical advice, or whom to send for. At this juncture the dentist prudently applied a bottle of chloroform to the nostrils with some relief, and when we arrived the patient was in a complete rigor, his chin quivering and his teeth chattering, as if chilled with cold and unable to speak. A bunch of carded cotton was sprinkled with chloroform, rolled in a kerchief, and applied around the neck, which was soon followed by relief; as soon as the patient could speak, he described the drawing of the muscles of the neck and face, the oppression at the chest, and his imperfect breathing as most excruciatingly painful. As soon as he was able, he was conveyed to his home, about a square's distance, when an anodyne was exhibited. Fearing a return of the symptoms, we insisted upon the extraction of the tooth, for which purpose the dentist was sent for, and it was extracted. Since then, he has had no return of the symptoms, which, from the subsequent relation he gave, were evidently tetanic.

The fomentation of the chloroform, and the small amount breathed into the lungs, acted most promptly and efficiently in controlling the spasm.

Case vi.—C——, a boy of nine years, was placed under our professional care for conjunctivitis. The history of the case showed that there was a proclivity to this disease, as he had suffered from previous attacks. He was placed under alteratives, and the lids of the eye closed by adhesive strips, and lint placed in the orbit and confined there by a bandage so as to exclude the light effectually. In a very short time the inflamation subsided, and the eye entirely recovered. There was at the time we first saw it, a nebulous formation at the margin of the iris, which was in an incredibly short time entirely removed.

Case vii.—Mrs. H——, an elderly lady of 65 years, had suffered for ten days from severe paroxysms of pain, beginning at 5 o'clock in the morning, lasting for an hour or two, during which the suffering was almost unendurable. The pain would begin simultaneously in the iliac and sacral regions, and then down the course of the ischiactic nerve of the right side.

The tongue was furred, the skin and eye icterode, appetite poor, the bowels constipated, the dejections clay colored, the urine scanty and colored almost a brown, bowels distended, and pain upon the gentlest pressure over the whole surface of abdomen, pain above the orbital arches, and coldness of the extremities particularly during paroxysms.

Diagnosed, a diurnal intermittent, complicating neuralgia of the sciatic nerve of one side, and those distributed over the abdominal parietal walls. Prescribed:

℞ Mass. Hyd., iij.,

Pulv. Opium, } aaj.

Pulv. Ipecac, }

Fiat pilula given at bed time, followed in the morning by

℞ Ol Ricini, ℥j.,

Ol Terebinth, ʒss. M

given at one dose. Should this move the bowels freely, then in three hours give one of the following powders, to be continued with the same intervals:

℞ Sulph. Quinia, ʒss.,
Pulv. Ipecac comp. grs. xv.,
M. Divide in Chart, no. xv.

This treatment was persisted in, during the three following days, with entire relief.

